# Digitalisierung für Gesundheit — ökonomische Aspekte des Gutachtens des SVR Gesundheit

**DOI:** 10.1007/s10273-021-2916-3

**Published:** 2021-05-17

**Authors:** Beate Jochimsen

**Affiliations:** FB Wirtschaftswissenschaften, Hochschule für Wirtschaft und Recht Berlin, Badensche Str. 50-51, 10825 Berlin, Deutschland

**Keywords:** I18, C80

## Abstract

Am 24. März 2021 hat der „Sachverständigenrat zur Begutachtung der Entwicklung im Gesundheitswesen“ sein Gutachten „Digitalisierung für Gesundheit — Ziele und Rahmenbedingungen eines dynamisch lernenden Gesundheitssystems“ der Bundesregierung überreicht. Wesentliche Aspekte des Gutachtens werden präsentiert, wobei ökonomische Gesichtspunkte und Bezüge zur Corona-Pandemie besonders hervorgehoben werden.

## Abstract (en)

When it comes to digitalising healthcare, Germany lags way behind other countries. In its 2021 report, the Health System Advisory Council discusses a strategy to overcome this shortcoming, i. e. the digitalisation of the German healthcare system. The idea is to shape digitalisation in a way that healthcare provision is improved with patients’ well-being in mind. Therefore, market failures in digital health markets have to be addressed as well as the chances and challenges that come with the usage of large amounts of data. Furthermore, this article highlights the potential of the electronic health record (EHR) and of the digital health app market and discusses requirements for reaching environmental and social sustainability within the context of the digitalisation of health.

Im internationalen Vergleich nimmt Deutschland bei der Digitalisierung des Gesundheitswesens keinen Spitzenplatz ein. Verbesserungsbedarf gibt es insbesondere bei informationstechnischen, organisatorischen und rechtlichen Aspekten in Bezug auf eine effiziente Versorgung sowie eine flächendeckende Implementierung des medizinischen Fortschritts. Ein Grund mag in der komplexen Struktur des deutschen Gesundheitswesens liegen. Das Prinzip der Selbstverwaltung, die Vielzahl der Träger stationärer und ambulanter Leistungen sowie der Föderalismus sorgen für eine Verflechtung der Verantwortlichkeiten, die oftmals nur schwer zu durchschauen ist und Innovationen, z. B. den Markteintritt digitaler Produkte, erschweren kann. Ein weiterer Grund liegt in der starken Regulierung dieses Bereichs, die sich zum einen aus verschiedenen Marktversagenstatbeständen ableitet, zum anderen aus einem derzeit sehr rigiden Verständnis von Datenschutz.

## Patientenwohl als Ziel der Digitalisierung

Da das Ziel einer jeglichen Maßnahme oder Neuerung im Gesundheitswesen stets das Patientenwohl ist, muss sich auch die Digitalisierung im Gesundheitswesen an diesem Maßstab messen lassen. Die Corona-Krise verdeutlicht, dass ihr eine Schlüsselrolle beim Erhalt des Patientenwohls, also beim optimalen Schutz von Leben und Gesundheit, zukommt. Beispiele hierfür wären Patientenwohlsteigerungen aufgrund einer Realtime-Erfassung der gesamten und freien Intensivbetten und Beatmungsplätze in Krankenhäusern, die Beschleunigung der Meldewege von Infektionsnachweisen und Genesungen an Gesundheitsämter und an das Robert Koch-Institut, die Ermittlung des Mobilitätsniveaus der Bevölkerung mit anonymisierten Mobiltelefondaten oder die Entwicklung und Nutzung einer leistungsfähigeren Corona-Warn-App (SVR Gesundheit, 2021, Z 46). Insbesondere bei den letzten beiden Punkten prallen verschiedene Normen des menschlichen Zusammenlebens aufeinander, nämlich das Recht auf informationelle Selbstbestimmung mit dem Recht auf freie Entfaltung der Person. Letzteres wäre in vielen Bereichen (Wirtschaft, Bildung, Kultur) gerade durch die Nutzung von (beispielsweise Mobilitäts-)Daten eher möglich.

Es gilt bei der Diskussion in Wissenschaft und Politik über die Digitalisierung im Gesundheitswesen alle betroffenen Normen in den Blick zu nehmen. Um das Patientenwohl zu maximieren, muss der Datenschutz neu gedacht werden. Er steht nicht isoliert, sondern muss mit dem Schutz von Leben und Gesundheit abgewogen werden. Informationelle Selbstbestimmung enthält nicht nur das Recht, dass die eigenen Daten geschützt werden, sondern ebenso, dass diese Daten zum eigenen Nutzen erhoben und verwendet werden dürfen. Statt Datenschutz sollte deshalb im Gesundheitssystem die Datensicherheit – mit harten strafrechtlichen Sanktionen im Falle eines Missbrauchs – zur Norm werden. Datensicherheit sieht eine sichere Nutzung von Gesundheitsdaten zur besseren Versorgung und Forschung vor. Damit kann das Wohl des Patienten unmittelbar steigen. Darüber hinaus ist zu diskutieren, inwiefern es in einer Gesellschaft zur Verantwortung jedes Individuums gehört, mit anderen solidarisch zu sein, indem es seine Gesundheitsdaten teilt. In einem – wie in

**Prof. Dr. Beate Jochimsen** ist Mitglied des Sachverständigenrats zur Begutachtung der Entwicklung im Gesundheitswesen und lehrt allgemeine Volkswirtschaftslehre, insbesondere Finanzwissenschaft, an der Hochschule für Wirtschaft und Recht Berlin.

Deutschland – solidarisch finanzierten Gesundheitssystem, kann es geboten sein, seine (unter starken Schutzgarantien pseudonymisiert auswertbaren) Gesundheitsdaten, etwa für kostenintensive Krebsbehandlungen, zur wissenschaftlichen Auswertung zur Verfügung zu stellen. Der Sachverständigenrat zur Begutachtung der Entwicklung im Gesundheitswesen meint: Daten teilen, heißt besser heilen.

Der Gesundheitsmarkt ist kein vollkommener Markt. Infolge des Konsums von Gesundheitsleistungen entstehen externe Effekte. Beispielsweise schützt eine SARS-CoV-2-Schutzimpung nicht nur den Impfling vor einer Erkrankung, sondern vermindert auch das Ansteckungsrisiko anderer. Es handelt sich somit um einen positiven externen Effekt. Darüber hinaus bestehen Informationsasymmetrien zwischen Angehörigen von Gesundheitsberufen und Patient:innen sowie zwischen beiden Gruppen und den Krankenversicherungen. Ferner erfolgt häufig eine Unterschätzung des Umfangs künftiger Erkrankungen, das Konsumentenverhalten ist also zeitinkonsistent (Oberender und Zerth, 2010). Schließlich herrscht auf Gesundheitsmärkten keine vollkommene Information. Transparenz von Qualität und Preisen ist vielfach nur rudimentär oder gar nicht vorhanden (Breyer, Zweifel und Kifman, 2013). Die Marktversagenstatbestände führen zu einer starken Regulierung des Gesundheitsmarkts und damit häufig zu einem Hemmnis bei der Implementierung innovativer digitaler Produkte in der Versorgung. Um die Potenziale der Digitalisierung in der Versorgung nutzen zu können, sollten „Innovationsoffenheit und die Bereitschaft zur regulatorischen Nachsteuerung“ die leitenden Prinzipien sein (SVR Wirtschaft, 2018).

## Besonderheiten digitaler Gesundheitsprodukte

Betrachtet man nicht den gesamten Gesundheitsmarkt, sondern spezielle Produkte, nämlich digitale Gesundheitsprodukte, weisen diese weitere Merkmale auf, die ein Marktversagen begründen können. Bei Entwicklung und Erstproduktion fallen häufig hohe Fixkosten an, bei Reproduktion und Vertrieb hingegen nur geringe, gegen null tendierende, variable Kosten (Urbach, 2017). Diese Kostenstruktur kann zu Skaleneffekten führen und damit das Entstehen natürlicher Monopole begünstigen. Verstärkt werden kann die Entwicklung hin zu natürlichen Monopolen durch eine hohe Intransparenz der Güterqualität. Vor dem Erwerb eines digitalen Gutes fällt es potenziellen Nutzer:innen häufig schwer, den Nutzen des Gutes bzw. der Anwendung zu beurteilen. Anders als bei vielen anderen Gütern wird der Nutzen bei digitalen Gütern auch durch die Zahl der Anwender (direkte Netzwerkeffekte) und das Vorhandensein von Komplementärangeboten (indirekte Netzwerkeffekte) bestimmt. Nutzen viele Individuen eine bestimmte Plattform, steigt die Wahrscheinlichkeit, dass darunter auch zahlreiche Leistungserbringer des Gesundheitssystems sind. Dadurch erhöht sich die Attraktivität der Nutzung dieser Plattform für den einzelnen Patienten, weil er erwarten kann, dass die Kommunikation und Koordination zwischen seinen Leistungserbringern und mit ihm leichter stattfinden kann (direkte Netzwerkeffekte: Frohmann, 2018).

Indirekte Netzwerkeffekte entstehen, sofern der Kauf eines digitalen Basisgutes (z.B. einer App zum Fruchtbarkeits-Tracking) durch das Angebot eines Komplementärgutes (z.B. Basalthermometer mit Bluetooth zur automatischen Übermittlung der Werte an die App) den Nutzen der entsprechenden Person erhöht. Für Unternehmen ist es oft attraktiver, ein Produktbündel zu produzieren, da sich Verbundvorteile ergeben können (Kommission Wettbewerbsrecht 4.0, 2019), und die Kundin die Kaufentscheidung zum Basisprodukt sofort (Upsell) oder später (etwa per In-App-Kauf) erweitern kann (SVR Gesundheit, 2021, Z 94). Ein Problem ist die häufig fehlende Interoperabilität von Produkten verschiedener Anbieter. Hohe Wechselkosten entstehen somit bei einem Anbieterwechsel, der deshalb trotz Qualitätsverschlechterungen oder Preissteigerungen eher unterbleibt. Dieses Phänomen bezeichnet man als Lock-in-Effekt (Urbach, 2017).

Ende 2020 gab es in den großen App Stores „Google Play“ und „App Store“ gut 90.000 Apps mit Gesundheitsbezug (Statista 2020a, 2020b). Trotz dieses reichhaltigen Angebots und großen Nutzenpotenzials sind sie bisher kaum in den ersten Gesundheitsmarkt, d.h. in den Erstattungsprozess der Gesetzlichen Krankenkassen, integriert. Auch im zweiten Gesundheitsmarkt, der alle privat finanzierten Gesundheitsprodukte und -dienstleistungen umfasst, spielen Gesundheits-Apps bisher keine große Rolle. In Deutschland nutzen sie lediglich 26 % der Bevölkerung regelmäßig (SVR Gesundheit, 2021, Z 323). Als Schlüsselelemente, um das Potenzial von Digitalen Gesundheitsanwendungen (DiGa) zu erschließen, gelten die Ermittlung ihrer tatsächlichen Effekte (Nutzenpotenzial) sowie ein anreizkompatibler Erstattungsprozess. Für eine gesundheitsökonomische Evaluation von DiGa könnte die Kosten-Nutzwert-Analyse (cost-utility analysis) herangezogen werden, die bei der Bewertung Lebensqualitäts- und Mortalitätseffekte in einer Größe (häufig quality-adjusted life years, QALY) ausdrückt und berücksichtigt. So wären Vergleiche verschiedener Interventionen/Apps über unterschiedliche Krankheitsbilder hinweg möglich. Der Gemeinsame Bundesausschuss (G-BA) lehnt diese international durchaus verbreitete Evaluationsform, vor allem die Verwendung von QALY, derzeit ab, weil sie als Vorbereitung für eine Rationierung anhand eines Schwellenwerts für ein gerade noch akzeptables Kosten-Nutzen-Verhältnis gesehen wird (SVR Gesundheit, 2021, Z 387).

Die Preise für DiGa bilden sich auf dem zweiten (d.h. privaten) Gesundheitsmarkt für Selbstzahlende ganz klassisch wie auf anderen Märkten für digitale Produkte. Dabei kann es zum

Angebot von Produktbündeln, aber auch zur Weiterverarbeitung personenspezifischer Daten als zusätzlicher Einnahmequelle kommen (Urbach, 2017). Im ersten Gesundheitsmarkt besteht die Herausforderung darin, Preise zu finden, die einerseits das solidarisch finanzierte Gesundheitssystem nicht überfordern und andererseits für den Anbieter ausreichende Anreize bieten, in die Entwicklung und Verbesserung von DiGa zu investieren. Derzeit gibt es neben einem festen Stückpreis die Möglichkeit, erfolgsabhängige Preisbestandteile zu gewähren (§ 134 Abs. 1 Satz 3 SGB V). Bei einer App zum Selbstmanagement bei einer chronischen Erkrankung könnten diese z.B. anhand der regelmäßigen Verwendung der App und damit der steigenden Wahrscheinlichkeit auf einen Therapieerfolg bemessen werden.

Die fehlende Interoperabilität innerhalb und zwischen Betriebssystemen und der damit einhergehende potenzielle Lock-in-Effekt sind derzeit fester Bestandteil des Geschäftsmodells der großen Internetplattformen bei DiGa. Solange der Vertrieb von Apps über die beiden großen Plattformen erfolgt, können diese ihre ohnehin vorhandene Marktmacht durch ihre Torwächter- und Regelsetzungsfunktion weiter ausbauen. Es gilt, den Wettbewerb in diesem Bereich zu sichern. Mit der Möglichkeit, hinsichtlich technischer Qualität gesicherte Gesundheits-Apps direkt über das Nationale Gesundheitsportal herunterladen zu können, wäre ein erster Schritt in diese Richtung getan (SVR Gesundheit, 2021, Z 158).

## Die elektronische Patientenakte (ePa)

Wie viele lebensrettende Hinweise hätten digital verfügbare Datensätze von Patient:innen in der Corona-Pandemie geben können? Wenn ein Abgleich potenzieller Risikofaktoren wie Übergewicht, Blutgruppe oder Vorerkrankungen digital und zeitnah möglich gewesen wäre? Wie viel besser hätte die medizinische Betreuung von Patient:innen mit COVID-19 sein können, wenn telemedizinische Unterstützung flächendeckend möglich gewesen wäre? Wie viel kontaktärmer und damit pandemieabschwächender wäre dies zudem gewesen? (SVR Gesundheit, 2021, Z 45). Eine elementare Form der ePa gibt es erst seit Januar 2021. Deshalb lagen diese Daten weder beim Ausbruch der Corona-Pandemie vor, noch liegen sie jetzt vor. Bei der momentan vorgesehenen Gestaltung der ePa werden sie auch noch lange nicht, vermutlich nie, vorliegen.

Bei einer ePa kann durch den zeitnahen und ortsunabhängigen Zugang zu strukturierten und konsistenten medizinischen Informationen eine bedarfsgerechte Versorgung erfolgen. Der SVR Gesundheit empfiehlt, eine ePa für jede Person mit Geburt oder Zuzug einzurichten und damit zugleich den Zugriff auf ePa-Daten für behandelnde Leistungserbringer zu ermöglichen. Jeder Person soll freistehen, der Einrichtung oder dieser Art von Zugriff zu widersprechen (Opt-out-Verfahren). Dies ist ein im internationalen Vergleich übliches Verfahren (vgl. Tabelle 1). Zudem sollte der oder die Versicherte entscheiden können, einzelne Inhalte der ePa für einzelne Leistungserbringer zu „verschatten“, sie also nicht zugänglich zu machen (SVR Gesundheit, 2021, Z 293).

Für den Erfolg der ePa in Deutschland wird von entscheidender Bedeutung sein, wie viele Personen sie nutzen. Nur bei einer hinreichend großen Zahl von Nutzer:innen lohnen sich zum einen Investitionen der Leistungserbringer in eine leistungsstarke Internetverbindung (z.B. um Röntgenbilder in der ePa abrufen zu können) sowie in sichere und effiziente Hard- und Software. Zum anderen werden sie nur dann routiniert mit der ePa umgehen. Die in Deutschland derzeit vorgesehene ePa erfordert separate und wiederholte Zustimmungen für Einrichtung, Zugriff zur Befüllung bzw. zur Einsichtnahme sowie für die Nutzung der Daten zu Forschungszwecken (mehrfaches Opt-in-Verfahren). Zu befürchten ist, dass aufgrund dieses großen und wiederholt auftretenden Aufwands, die Chancen und Potenziale der ePa von zu wenigen genutzt werden (SVR Gesundheit, 2021, Z 294).

## Finanzierung der Digitalisierung

Die Gesundheitsausgaben in Deutschland betrugen 2018 rund 12 % des Bruttoinlandsprodukts (391 Mrd. Euro), wovon jedoch nur 2 % (7 Mrd. Euro) auf Investitionen (auch für Digitalisierung) entfielen (Destatis, 2020a). Knapp 60 % der Ausgaben trug die gesetzliche Krankenversicherung (GKV), 13 % die privaten Haushalte und private Organisationen ohne Erwerbszweck, gefolgt von der Pflegeversicherung mit 10 % und der Privaten Krankenversicherung mit 8,5 %. Auf die öffentlichen Haushalte entfielen 4 % der Ausgaben (Destatis, 2020b). Die finanziellen Auswirkungen der Digitalisierung im Gesundheitswesen sind derzeit praktisch nicht abschätzbar. Digitalisierung führt erst einmal zu hohen zusätzlichen Ausgaben in Infrastruktur und Kompetenzaneignung, später zu Einsparungen z.B. infolge wegfallender Doppeluntersuchungen und zielgerichteter Behandlung. Inwiefern die zusätzlichen Ausgaben die zu erwartenden Einsparungen übersteigen ist offen. Im regulierten deutschen Gesundheitssystem werden Investitionen in Digitalisierungsprojekte sowie die Finanzierung der laufenden Kosten von informationstechnischen Systemen zum Großteil mit den bestehenden Finanzierungsinstrumenten getätigt. Dazu zählen die Beitragsmittel der GKV, Steuermittel von Bund und Ländern (teilweise kofinanziert durch die EU) sowie Investitionen Privater im stationären und ambulanten Bereich (SVR Gesundheit, 2021, Z 145). Mit dem Krankenhauszukunftsfonds stellt der Bund beispielsweise für 2021 bis 2023 insgesamt 3 Mrd. Euro zur Verfügung, die explizit für die Förderung der digitalen Ausstattung der Krankenhäuser vorgesehen sind und mit zusätzlich 1,3 Mrd. Euro von den Ländern kofinanziert wer-

Tabelle 1

**Einwilligungsverfahren zur Nutzung elektronischer Patientenakten (ePA) in sechs ausgewählten Ländern**

| | Dänemark | England | Estland | Österreich | Schweden | Spanien |
|---|---|---|---|---|---|---|
| Einwilligungsverfahren ePA allgemein[1] | Standard | Opt-out | Opt-out | Opt-out | Kurzakte: Opt-in, regionale ePA: Standard | Kurzakte: Standard (kein Opt-out möglich) |
| Einwilligunsverfahren Einsichtnahme durch LE[2] | Opt-out | Kein separates Einwilligungsverfahren | Opt-out | Feingranulares Opt-out | Kurzakte: Feingranulares Opt-out | Feingranulares Opt-out |
| Nutzung zu Forschungszwecken möglich? | Grundsätzlich möglich | Opt-out | Grundsätzlich möglich | Derzeit nein | Keine Kurzakte, aber hoher Anteil an Qualitätsregistern, z. T. in regionale ePA integriert | Opt-in |
| Zahl ePA-Nutzende der Bevölkerung | Regional verschieden, sundhed.dk: 1,7 Mio./Monat | >96 % der Bevölkerung | 99 % der Bevölkerung | 97 % der Bevölkerung | ePA: fast die gesamte Bevölkerung | 95 % der versorgten Bevölkerung |

Anmerkungen: 1 Das Einwilligungsverfahren zur ePA wird in drei Kategorien unterteilt: *Standard*: Jede Person erhält eine ePA, in der die Dienstleister des Gesundheitswesens die medizinischen Informationen einspeisen. *Opt-out*: Jede Person erhält ebenfalls eine ePA, in der die Dienstleister des Gesundheitswesens die medizinischen Informationen einspeisen. Allerdings besteht die Möglichkeit für Patient:innen, der Einrichtung und der Führung einer solchen Akte zu widersprechen. Bezogen auf die Einsichtnahme haben Patient:innen die Möglichkeit, auf Dokumenten-Ebene (feingranular) oder bezogen auf Leistungserbringer der Einsichtnahme durch Behandelnde zu widersprechen. *Opt-in*: Jede Person erhält eine ePA, wenn sie aktiv zustimmt. Bezogen auf die Einsichtnahme müssen Patient:innen bei einem Opt-in-Verfahren erst ihre Einwilligung zur ePA-Einsicht erteilen. 2 LE: Leistungserbringende.

Quelle: eigene Darstellung des SVR Gesundheit (2021, 104, 99).

den sollen. Die GKV finanziert etwa einen Teil der Digitalisierung im ambulanten Bereich durch pauschale Erstattungsbeträge für Hardwareinvestitionen und Softwareleistungen. Ebenso wenig wie die insgesamt durch die GKV zu tragenden Kosten der Digitalisierung im Gesundheitswesen sind die gesamtwirtschaftlichen Kosten derselben abzuschätzen. Fragen nach Kosten-Nutzen-Abwägungen/Analysen vor Einführung bestimmter digitaler Elemente werden de facto nicht gestellt.

Gerade aufgrund dieser komplexen Gemengelage ist es wichtig, eine effiziente, anreizkonforme und transparente Finanzierung sicherzustellen. Bei der Finanzierung im stationären Sektor sollte darauf geachtet werden, die Digitalisierung zu fördern, ohne dabei ineffiziente Krankenhausstrukturen zu festigen. Das kann geschehen, indem die z. B. durch den Krankenhauszukunftsfonds finanzierten Investitionen an Strukturveränderungen geknüpft werden. Langfristig bietet sich eine Übernahme der Investitionskosten im Digitalisierungsbereich in die Regelfinanzierung an, vorzugsweise parallel zu einem Übergang zu einer monistischen Krankenhausfinanzierung (SVR Gesundheit, 2018).

## Ökologische und soziale Nachhaltigkeit

Für eine effektive Digitalisierung des Gesundheitswesens ist eine vermehrte Datenerzeugung und -speicherung unabdingbar. Damit geht ein erhöhter Strombedarf zum Betrieb einer größeren Zahl technischer Geräte einher, der – je nach Energieerzeugungsmethode – zu einem vermehrten Schadstoffausstoß führen kann. Ferner wird es einen gesteigerten Bedarf an Rohstoffen wie Lithium oder Seltenen Erden geben, der eine Übernutzung dieser sowie Gesundheitsschäden beim Abbau verursachen kann. Standards für menschenwürdige Arbeitsbedingungen entlang der Lieferkette sowie für Energieeffizienz, Recyclingvorgaben und Langlebigkeitsanreize für Produkte können politische Maßnahmen sein, um dem Ziel der ökologischen Nachhaltigkeit näherzukommen. Sofern sich diese Maßnahmen nicht kostenneutral umsetzen lassen, müssten höhere Kosten in Kauf genommen werden (SVR Gesundheit, 2021, Z 159).

Gleichzeitig gilt es, soziale Nachhaltigkeit zu erreichen, d. h. in diesem Zusammenhang gesundheitliche Ungleichheiten in Bezug auf den Gesundheitszustand und die Gesundheitsversorgung, die aufgrund sozioökonomischer Charakteristika vorherrschen, zu mindern. Bestimmte Personengruppen partizipieren weniger am digitalen Wandel und profitieren folglich weniger von neuen digitalen Versorgungsmöglichkeiten. So haben Alter, Geschlecht, Bildungsstand und finanzielle Ressourcen ebenso einen Einfluss auf die Inanspruchnahme digitaler Gesundheitstechnologien (Azzopardi-Muscat und Sorensen, 2019) wie geografische Ungleichheit infolge variierender Infrastruktur. Vergleicht man die Datenübertragungsraten in Deutschland, so ergeben sich sowohl Unterschiede zwischen west- und ostdeutschen Bundesländern als auch zwischen städtischen und ländlichen Gebieten und zwar sowohl für die privaten Zugänge als auch für die Zugänge der stationären Versorgung. Infrastrukturelle Unterschiede zeigen sich auch hinsichtlich sozioökonomischer Merkmale. So sinkt die Verfügbarkeit über ein Smartphone sowie die Nutzung eines Computers mit dem Alter, vor allem ab 65 Jahren (SVR Gesundheit, 2021, Z 90, Z 92). Die Sicherstellung eines flächendeckenden Zugangs zu einer leistungsfähigen Breitbandstruktur und zur notwendigen

Hard- und Software für alle Bevölkerungsgruppen und Leistungserbringer ist somit unabdingbar.

Ebenso hängt die Bereitschaft, zukünftig eine ePa zu nutzen, signifikant vom Geschlecht und Bildungsstand ab. Derzeit nutzen jüngere Männer mit höherem Bildungsstand eine elektronische Gesundheitsakte häufiger als Frauen, ältere Menschen oder solche mit mittlerem oder niedrigem Bildungsstand. Ähnliche Beobachtungen liegen für die Nutzung von Gesundheits-Apps vor, die von jüngeren Menschen mit höherer Bildung öfter genutzt werden (Cornejo Müller et al., 2020). Gerade diese Menschen bewerten auch den Nutzen von Gesundheits-Apps höher, was die Ungleichheit der Nutzung und des daraus resultierenden Nutzens künftig noch verstärken könnte (SVR Gesundheit, 2021, Z 623). Vor diesem Hintergrund begrüßt der SVR Gesundheit die im Nationalen Aktionsplan Gesundheitskompetenz dargelegten strukturellen und programmatischen Förderungsmaßnahmen, um die entsprechende Kompetenz nicht nur bei den Bürger:innen, sondern auch bei den Angehörigen der Heilberufe zu festigen und zu erhöhen. Darüber hinaus sollte ein besonderes Augenmerk auf die gezielte Förderung benachteiligter Bevölkerungsgruppen angestrebt werden.

## Fazit

Die Digitalisierung kann die Gesundheitsversorgung verbessern und das Patientenwohl steigern. Um diese Ziele zu erreichen, müssen die notwendigen infrastrukturellen und ökonomischen Rahmenbedingungen geschaffen werden. Dazu zählen unter anderem der Ausbau des Glasfasernetzes, die zeitnahe Implementierung der nötigen Hard- und Software (z. B. bei der Telematik), eine verbindliche und international anschlussfähige Interoperabilität (z. B. bei der ePa) sowie die Sicherung des Wettbewerbs trotz der Besonderheiten des Gesundheitsmarkts und der -produkte. Mit der Digitalisierung geht die Generierung von Daten einher, deren verantwortungsvolle Auswertung zu Forschungszwecken den Nutzen der heutigen und künftigen Patient:innen steigern kann. Um die Bereitschaft der Menschen, ihre Daten zu Forschungszwecken zur Verfügung zu stellen, und ihr Vertrauen beispielsweise in die ePa zu steigern, müssen höchste Anforderungen an Daten- und Informationssicherheit sämtlicher digitaler Technologien im Gesundheitswesen gelten. Statt Datenschutz sollte somit Datensicherheit zur Norm werden. Im Sinne einer sozialen Nachhaltigkeit gilt es, Ungleichheit aufgrund sozioökonomischer Charakteristika zu mindern. Dafür ist die Förderung der digitalen Gesundheitskompetenz der gesamten Bevölkerung ebenso notwendig wie die gezielte Unterstützung benachteiligter sozialer Gruppen. Schließlich fordert der SVR Gesundheit, die Digitalisierung des Gesundheitswesens in eine umfassende Nachhaltigkeitsstrategie einzubetten (SVR Gesundheit 2021, Z 30).

**Title:** ***Digitalisation for Health – Some Economic Aspects***

**JEL Classification:** *I18, C80*
